# Use of Smart Technology for the Early Diagnosis of Complications After Cardiac Surgery: The Box 2.0 Study Protocol

**DOI:** 10.2196/16326

**Published:** 2020-04-21

**Authors:** Tom E Biersteker, Mark J Boogers, Robert AF de Lind van Wijngaarden, Rolf HH Groenwold, Serge A Trines, Anouk P van Alem, Charles JHJ Kirchhof, Nicolette van Hof, Robert JM Klautz, Martin J Schalij, Roderick W Treskes

**Affiliations:** 1 Department of Cardiology Leiden University Medical Center Leiden Netherlands; 2 Department of Cardiothoracic Surgery Leiden University Medical Center Leiden Netherlands; 3 Department of Clinical Epidemiology Leiden University Medical Center Leiden Netherlands; 4 Department of Cardiology Haaglanden Medisch Centrum Den Haag Netherlands; 5 Department of Cardiology Alrijne Ziekenhuis Leiderdorp Netherlands

**Keywords:** mHealth, cardiac surgery, atrial fibrillation, postoperative care, ambulatory electrocardiography

## Abstract

**Background:**

Atrial fibrillation (AF), sternal wound infection, and cardiac decompensation are complications that can occur after cardiac surgery. Early detection of these complications is clinically relevant, as early treatment is associated with better clinical outcomes. Remote monitoring with the use of a smartphone (mobile health [mHealth]) might improve the early detection of complications after cardiac surgery.

**Objective:**

The primary aim of this study is to compare the detection rate of AF diagnosed with an mHealth solution to the detection rate of AF diagnosed with standard care. Secondary objectives include detection of sternal wound infection and cardiac decompensation, as well as assessment of quality of life, patient satisfaction, and cost-effectiveness.

**Methods:**

*The Box 2.0* is a study with a prospective intervention group and a historical control group for comparison. Patients undergoing cardiac surgery at Leiden University Medical Center are eligible for enrollment. In this study, 365 historical patients will be used as controls and 365 other participants will be asked to receive either *The Box 2.0* intervention consisting of seven home measurement devices along with a video consultation 2 weeks after discharge or standard cardiac care for 3 months. Patient information will be analyzed according to the intention-to-treat principle. *The Box 2.0* devices include a blood pressure monitor, thermometer, weight scale, step count watch, single-lead electrocardiogram (ECG) device, 12-lead ECG device, and pulse oximeter.

**Results:**

The study started in November 2018. The primary outcome of this study is the detection rate of AF in both groups. Quality of life is measured with the five-level EuroQol five-dimension (EQ-5D-5L) questionnaire. Cost-effectiveness is calculated from a society perspective using prices from Dutch costing guidelines and quality of life data from the study. In the historical cohort, 93.9% (336/358) completed the EQ-5D-5L and patient satisfaction questionnaires 3 months after cardiac surgery.

**Conclusions:**

The rationale and design of a study to investigate mHealth devices in postoperative cardiac surgery patients are presented. The first results are expected in September 2020.

**Trial Registration:**

ClinicalTrials.gov NCT03690492; http://clinicaltrials.gov/show/NCT03690492

**International Registered Report Identifier (IRRID):**

DERR1-10.2196/16326

## Introduction

Over the past decades, major advances have been made to improve the safety of cardiac surgery in order to decrease the risk of adverse events [1]. However, a series of life-threatening events can occur after cardiac surgery. Even patients who are discharged in a clinically stable condition are still at risk for the development of complications at home. The most frequently occurring postoperative complications are cardiac decompensation, late tamponade, and rhythm disturbances, such as atrial fibrillation (AF). In approximately 25% of all patients, one or more of these complications occur, and they are mostly noted in the ward at day 2 or 3 after surgery [2-4]. At day 7 or later, postoperative atrial fibrillation (POAF) is diagnosed in approximately 15% of all cases [5]. Sternal wound infection occurs in 3% to 5% of cases, with mediastinitis occurring in 1% to 2% of all cases [6-8]. Late-onset sternal wound infection is defined as sternal wound infection occurring 14 days or later after the initial surgery, and it is relatively as frequent as early-onset sternal wound infection [9].

Early detection of these complications is of vital importance as there can be various issues (eg, untreated AF is associated with an increased risk of transient ischemic attack and ischemic stroke) [10,11]. Moreover, with early diagnosis of cardiac decompensation or wound infection, hospital readmission may be prevented [12]. Previous research has shown that patients do not always recognize complications at home, which may delay the diagnosis and thus cause more risk and harm to the patient [13]. Telephone calls as a follow-up strategy have been found to be helpful, but ambulatory diagnosis of postoperative complications remains challenging [14].

Advances in information and communication technologies have led to more possibilities for monitoring patients remotely (telemonitoring) and using a smartphone to provide medical care (mobile health [mHealth]) [15]. For example, smartphone-compatible detectors for cardiovascular disease parameters, such as Kardia (AliveCor Inc, San Francisco, California, USA) and Withings Blood Pressure Monitor (Withings, Issy les Moulineaux, France), have been released in the consumer market [16-19]. These devices are capable of measuring blood pressure (BP), blood oxygen saturation, weight, temperature, and number of daily steps taken, as well as electrocardiogram (ECG) registration, providing patients with the possibility to monitor their vital parameters at home.

In addition to improved monitoring, mHealth may have more benefits. Video conferences between doctors and patients may save time and money for patients, especially in rural areas [20,21]. Unpublished results of *The Box*, an mHealth study in patients after myocardial infarction, which has been registered under clinical trial number NCT02976376 at ClinicalTrials.gov, suggest that remote follow-up is cost-effective [22].

It is hypothesized that smart technology can help with the early diagnosis of late complications and thereby improve quality of care and patient satisfaction after cardiac surgery. The aim of this study is to investigate the clinical effectiveness of a smart technology intervention in patients after cardiac surgery. The rationale and design of this study are presented.

## Methods

### Study Design

*The Box 2.0* is a study with a prospective intervention group and a historical control group for comparison. The study is being conducted at the Department of Cardiothoracic Surgery and Department of Cardiology in Leiden University Medical Center (LUMC), a tertiary care hospital in Leiden, The Netherlands. The study is registered at ClinicalTrials.gov under trial number NCT03690492 and registered at Toetsingonline.nl under number NL65959.058.18, and has been approved by the Medical Ethics Committee of LUMC (P18.110). All procedures are conducted in accordance with the principles of the Declaration of Helsinki (version 10, October 2013) and in accordance with the Dutch Medical Research Involving Human Subjects Act (Wet Medisch-wetenschappelijk Onderzoek met mensen) and Good Clinical Practice. Written offline informed consent will be obtained from all prospective study participants.

### Patient Population

Adult patients undergoing coronary artery bypass grafting, valve reconstruction or replacement, surgery of the aortic root or ascending aorta, or other cardiac surgeries performed via median sternotomy, such as atrial or ventricular septal defect closure, a Dor or Morrow procedure, cardiac tumor removal, and surgical treatment of coronary artery anomalies, are eligible for enrolment. Patients with perioperative endocarditis, those with a need for mechanical support with extracorporeal membrane oxygenation (ECMO), an Impella device, or an intra-aortic balloon pump (IABP), and those with an Interagency Registry for Mechanically Assisted Circulatory Support (INTERMACS) score of 1 or 2 are excluded. All inclusion and exclusion criteria are listed in Textbox 1.

**Study inclusion and exclusion criteria.**

**Inclusion criteria**

- Patient undergoes cardiac surgery (coronary artery bypass grafting, valve reconstruction or replacement, aortic root or ascending aortic surgery, or other cardiac surgeries performed via median sternotomy, such as atrial or ventricular septal defect closure, a Dor or Morrow procedure, cardiac tumor removal, and surgical treatment of coronary artery anomalies.
- Patient is able to communicate and is literate in English or Dutch.

**Exclusion criteria**

- Patient is less than 18 years old.
- Patient is pregnant.
- Patient is considered an incapacitated adult.
- Patient is unwilling to sign the informed consent form.
- Patient undergoes emergency cardiac surgery (Interagency Registry for Mechanically Assisted Circulatory Support score 1 or 2).
- Patient has active endocarditis at the time of operation.
- Patient is on mechanical circulatory support before operation.
- Patient has a ventricular septal rupture.
- Patient undergoes extracorporeal membrane oxygenation or ventricular assist device insertion.

### Patient Selection

The historical cohort consists of patients who underwent cardiac surgery at LUMC from January to September 2018. Among the patients, 93.9% (336/358) completed the five-level EuroQol five-dimension (EQ-5D-5L) questionnaire and patient satisfaction questionnaire 3 months after cardiac surgery. These questionnaires are consistent with the questionnaires to be filled out by prospective study patients.

The prospective cohort is selected from November 2018 onwards. Patients eligible for participation are recruited through three different pathways as follows: patients who are electively awaiting cardiac surgery will be approached for participation in the outpatient clinic 4 to 6 weeks before their operation; patients who are admitted to the cardiology ward and awaiting surgery will be approached for participation approximately 3 to 5 days before surgery; and patients from a referring hospital who are transferred to the cardiothoracic surgery ward of LUMC awaiting surgery will be approached for participation approximately 2 days before surgery. If a patient is not included in the study preoperatively, the patient can be asked to participate after the operation until discharge or transfer back to the referring hospital.

### Standard Postoperative Care

Fourteen days and 3 months after cardiac surgery, patients are seen by a nurse practitioner (NP), who is supervised by a consultant cardiologist. At 14 days, the sternal wound and, if applicable, the venous graft resection site are checked, and vital parameters, signs of congestion, indications for and side effects of medications, and symptoms of postoperative complications are assessed.

At 3 months, a similar examination is performed during an outpatient clinic visit. In addition, blood samples are taken for measuring kidney function and lipid spectrum. Moreover, a transthoracic echocardiogram is obtained. Patients undergoing a mini-Maze procedure or concomitant radiofrequency ablation will also undergo 24-hour Holter monitoring and physical exercise testing at 3 and 12 months after surgery.

### The Box 2.0

#### The Box 2.0 Intervention

**Figure 1:**
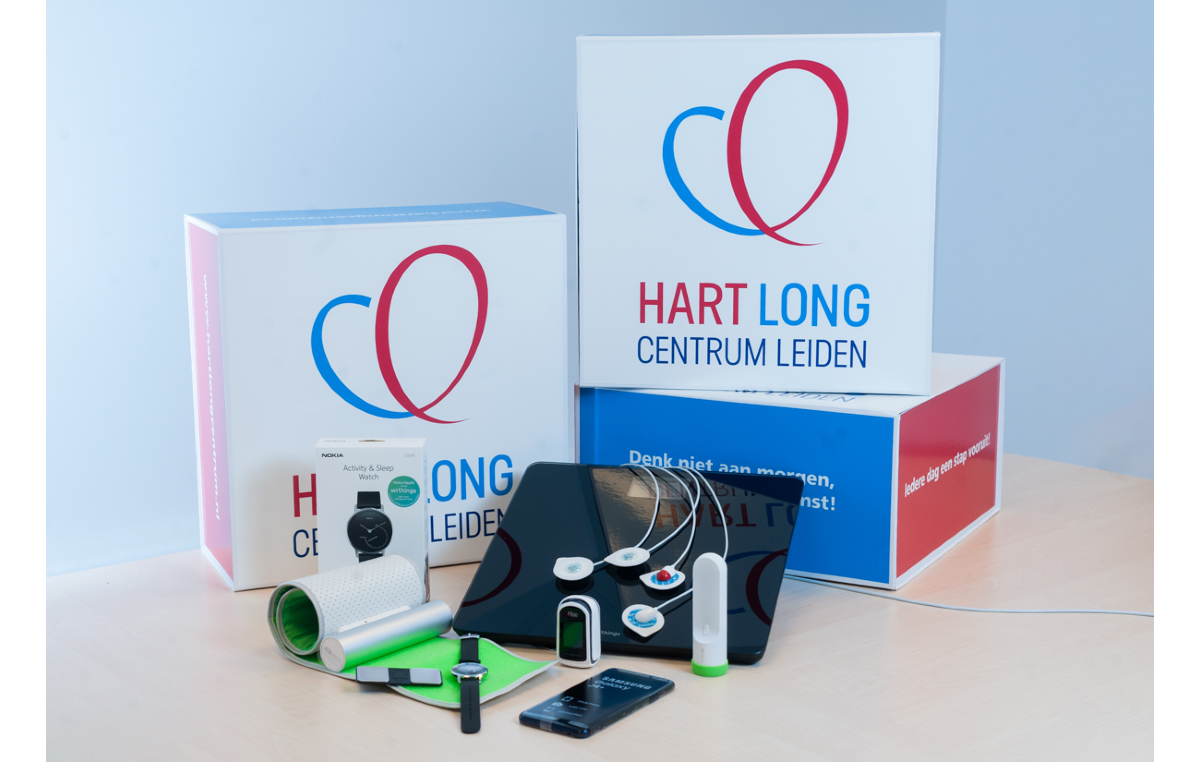
The devices of *The Box 2.0*.

Patients who consent to participate in *The Box 2.0* will receive a box containing a Withings weight scale, Withings BP monitor, Withings activity tracker, Withings thermometer, blood oxygen saturation monitor (Masimo, Irvine, California, USA), and two mobile ECG devices (AliveCor Kardia and CardioSecur; Personal MedSystems, Frankfurt am Main, Germany). The devices are shown in Figure 1.

All are noninvasive, battery-powered, smartphone-compatible devices. They are Conformité Européenne marked and approved by the Food and Drug Administration and can be obtained in retail stores in the European Union and the United States. No assistance is required to install and use the devices; however, professional assistance is provided by a technical LUMC employee when a patient requires help.

A smartphone with iOS (Apple Computers, Cupertino, California, USA) or Android Operating System (Google, Mountain View, California, USA) is required to use the devices, as they communicate with a dedicated mobile app on the smartphone, which can be downloaded from the AppStore (iOS) or PlayStore (Android). Measurement data are stored on the smartphone and uploaded to the app manufacturer’s servers (located in Europe for Withings and CardioSecur and the United States for AliveCor and Masimo). An internet connection is required to synchronize the data, although measurements can be obtained when the smartphone is offline. The data are uploaded to the corresponding servers when the smartphone reconnects to the internet.

Patients receive *The Box 2.0* before discharge. A technical support employee of LUMC instructs the patients on how to install and use the devices on their mobile phones, including registering necessary accounts. This employee also manages a help desk service that patients can reach for technical issues with their devices. Patients are also provided with written information and an instruction video. Patients who do not own a smartphone with either iOS or Android are provided with a Samsung J3 smartphone (Samsung, Seoul, South Korea). Patients are instructed to use their own internet service (WiFi or mobile network). No mobile data network plan is provided with the smartphone. Additionally, to be able to use the accounts, an email address is required. Patients are provided with a randomly generated email address from the @hlc.nl domain, which is owned and maintained by LUMC.

Patients are instructed to record their BP, weight, temperature, step count, blood oxygen saturation, respiration rate, and single-lead ECG data with AliveCor Kardia on a daily basis for the first 14 days. After 14 days, all measurements except for step count are reduced to thrice a week. Additionally, patients record a CardioSecur ECG weekly. In case of a possible rhythm disturbance, which is either felt by the patient or diagnosed with the single-lead ECG, patients obtain an additional CardioSecur ECG.

Moreover, the first visit to the outpatient clinic is replaced by an electronic visit, in which the patient communicates with the NP via Webcamconsult (Webcamconsult, Bergen op Zoom, The Netherlands), a secured video connection. The same topics as those during a regular visit are discussed, and the sternal wound is checked. Therefore, patients are advised to wear a dress shirt when establishing the video connection, so that no private body parts are shown.

**Figure 2:**
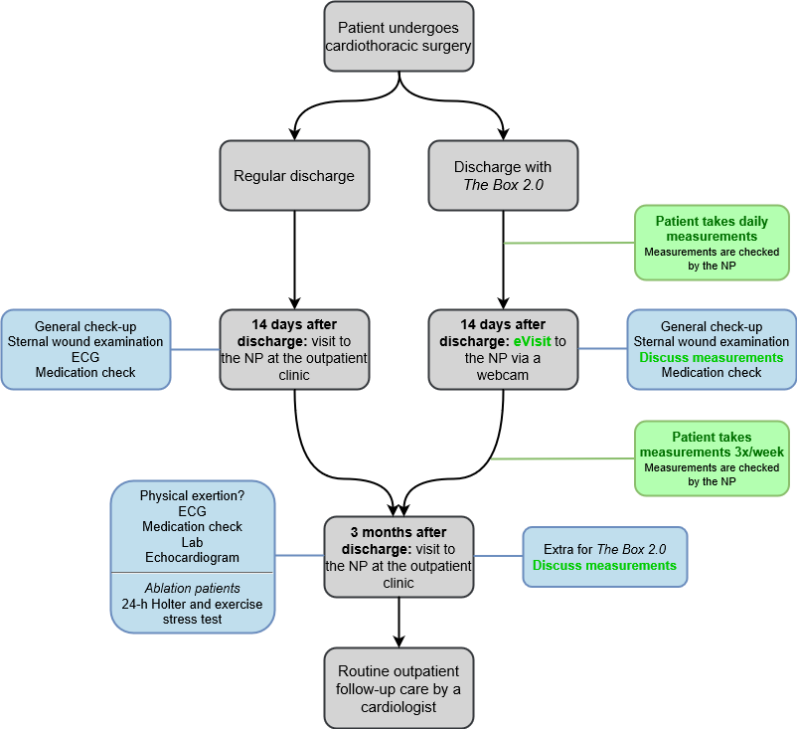
Flowchart for the outpatient clinic follow-up. Left: standard outpatient care without the mHealth intervention. Right: outpatient care with the mHealth intervention. Blue text boxes show the standard topics at 14-day and 3-month consultations. Green text boxes and text highlight the mHealth intervention. ECG: electrocardiogram; NP: nurse practitioner.

An overview of regular follow-up and follow-up with *The Box 2.0* is shown in Figure 2.

#### Blood Pressure Monitor

The BP monitor (Withings Blood Pressure Monitor) is a validated [23] smartphone-compatible device that allows users to measure systolic and diastolic BP and heart rate. Depending on user preference, the device is placed around the left or right arm, and after pushing a button on the cuff, the device connects to the smartphone via Bluetooth. A measurement takes an average of 20 seconds, and inflation and deflation are automated and initiated via the dedicated Health Mate app for iOS and Android. The results are shown on the smartphone screen and automatically uploaded to Withings servers.

#### Weight Scale

The weight scale (Withings Body) measures weight, fat percentage, heart rate, and ambient CO_2_. By footprint recognition, all measurements are automatically saved under each user’s personal account. This way, measurements of users cannot be confused. The results are shown on the weight scale screen and automatically uploaded to Withings servers.

#### Thermometer

The thermometer (Withings Thermo) is able to measure the temperature of the temporal artery by scanning the forehead. Additionally, a measurement of the dorsal auricular artery can be obtained by placing the device on the soft tissue underneath the ear. The highest measurement will be used. The results are shown on the thermometer and automatically uploaded to Withings servers.

#### Activity Tracker

The activity tracker (Withings Steel) automatically tracks the number of daily steps taken and the duration and quality of sleep. The device is a small watch and can also be used as such. Results are automatically uploaded to Withings servers.

#### Blood Oxygen Saturation Monitor

The oxygen saturation monitor (Masimo MightySatRx) tracks blood oxygen saturation, as well as heart rate, respiration rate, plethysmography variability index, and peripheral perfusion index. The device is placed on a finger, and measurements start automatically after starting the app. Masimo claims that its device and algorithm work on well-circulated and less well-circulated fingers. Measurements are automatically saved to a comma-separated file on the smartphone. This file can then be shared by email. No measurement data are saved on Masimo servers.

#### Single-Lead Electrocardiogram Device

The single-lead ECG device (AliveCor Kardia) uses two electrodes to allow a user to record a 30-second lead-I ECG. The device connects to the AliveCor app via an ultrasound signal, and to record an ECG, the patient places two fingers of both hands on the electrodes while the device is held next to the smartphone at a maximum of 30 cm. The device can also be attached to the back of the smartphone.

The ECG signal is converted to a live single-lead ECG that is shown on the smartphone screen [24]. After the measurement is completed, the AliveCor algorithm calculates the R-R intervals and reports either a normal ECG, possible AF, or undetermined finding [16]*.* Before saving the ECG, the patient can save notes. The algorithm has a 70% to 97% sensitivity and 98% to 99% specificity for the detection of AF [25-27].

#### EASI-Derived 12-Lead Electrocardiogram Device

The CardioSecur ECG device uses the five-electrode EASI lead system described by Dower et al [28], removing the ground electrode. It is hypothesized to detect myocardial ischemia and have an amount of baseline noise like that of Mason-Likar ECG [17]. This possibly allows for relatively noise-free rhythm detection. As no external validation studies have been performed, this study will not use the CardioSecur device for the detection of myocardial ischemia.

Patients receive their first CardioSecur ECG in the hospital preoperatively. The four leads are connected to the chest of the patient. One lead is placed on the upper part of the sternum, one lead is placed directly underneath the sternum, and two leads are placed on the mid-axillary line at the same height as that of the lower placed frontal lead. After registration of the first ECG, patients can take their own ECG at home. The ECG is uploaded to CardioSecur servers and sent to the hospital electronic medical record in portable document format (PDF).

**Figure 3:**
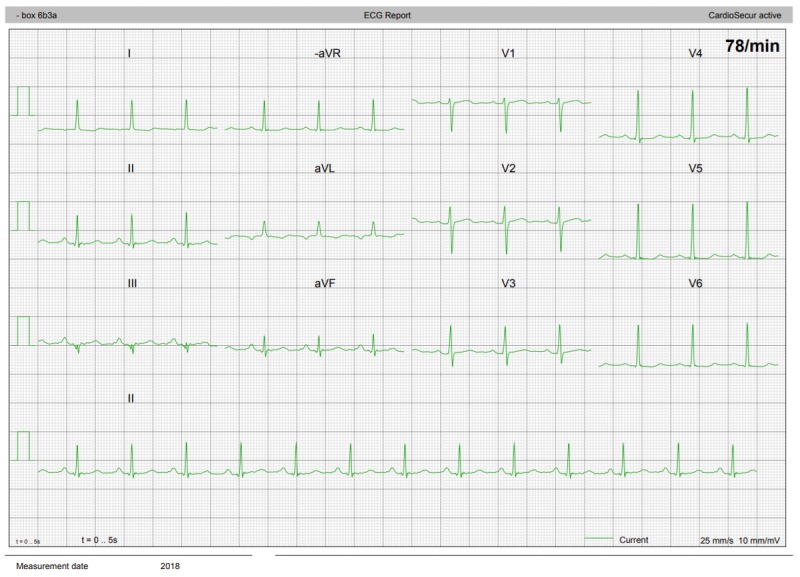
An electrocardiogram obtained with the CardioSecur device.

**Figure 4:**
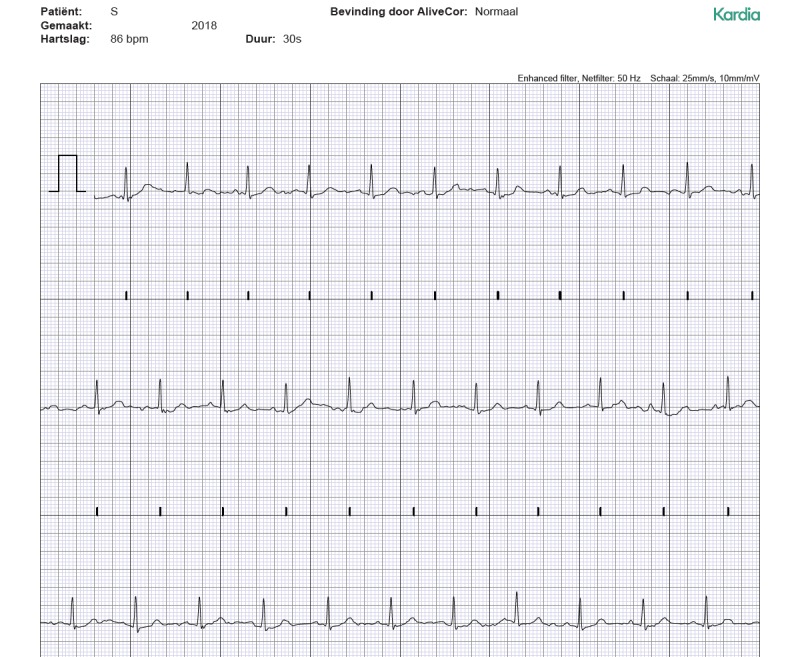
A lead-I electrocardiogram obtained with the AliveCor Kardia device.

Figures 3 and 4 show examples of an ECG obtained with CardioSecur and a single-lead ECG obtained with AliveCor Kardia, respectively.

### Reasons to Contact Patients

The sent-in data are checked daily by a dedicated NP. Data irregularities trigger an automated alarm. When irregularities are noted, patients are contacted within 48 hours after the data have been sent in. The definitions of data irregularities are shown in Multimedia Appendix 1.

### Reasons to Adjust the Therapeutic Regimen

According to the sent-in data, the NP can change medication or discuss the data with the patient’s treating physician. Detecting an atrial rhythm disturbance with the CardioSecur may thus result in adjusting the therapeutic regiment. This is left to the discretion of the treating physician.

### Nonadherence and Study Withdrawal

Patients are required to frequently send in their measurements. If they do not send measurements from their devices for 21 consecutive days, they are considered nonadherent. This is the same policy as in the previous *The Box* study [22]. If a patient is considered nonadherent, a standardized email is sent, urging them to contact the hospital in case of technical difficulties or objections. A specialized technology employee can provide assistance. If, after this phone call, the patient starts sending in measurements, the patient is again considered adherent. If nonadherence reoccurs, a standardized email is sent out. If patients do not start sending in new measurements, they are not approached again and are considered definitively nonadherent. These patients are not excluded from the study and are still followed up according to *The Box 2.0* protocol. If any study patients notify the hospital that they would like to have regular outpatient clinic visits, they are followed up via the regular follow-up protocol. Patients who withdraw from regular follow-up are considered lost to follow-up.

### Privacy of Study Participants

To anonymize the data, patients receive a randomly generated email address from the @hlc.nl domain, which is owned and maintained by LUMC. They can use this email address to create their Withings and CardioSecur accounts, as well as send the comma-separated file (created by using the Masimo device) to LUMC. The corresponding names are kept in a separate password-protected database.

## Results

### Outcomes

The study started recruitment in November 2018. The primary outcome measure of this study in patients undergoing cardiac surgery is to investigate if POAF is diagnosed more often in patients who are followed up with smart technology as compared with standard care, with measurements until the 3-month outpatient clinic visit. POAF can be diagnosed before or after discharge. As this mHealth study does not impact the AF detection rate before discharge, the primary outcome only involves POAF diagnosed after discharge. In LUMC, the mean duration of hospital stay after cardiac surgery is 8 days. POAF diagnosed before discharge is reported as a baseline characteristic.

AF is defined as an episode of irregular heart rhythm, without detectable P waves, lasting more than 30 seconds [29]. AF can be detected at the outpatient clinic, at the emergency department, or at home via CardioSecur in *The* *Box 2.0* patients. As taking an ECG with CardioSecur takes longer than 30 seconds, we defined POAF or atrial flutter as a CardioSecur ECG that shows this rhythm disturbance.

The primary endpoint is evaluated by a clinical event committee that is blinded to patient data. The committee consists of two cardiologists, not otherwise involved in the project, who review the data independently. In case of disagreement, a third cardiologist is involved to reach a decision.

Secondary outcomes of this study include the diagnosis of cardiac decompensation or sternal wound infection. Sternal wound infection is defined by the guidelines of the Center for Disease Control and Prevention, which are most commonly used. For deep sternal wound infection, fever (temperature >38.5°C), sternal instability, or chest discomfort has to be present in combination with either purulent drainage from the sternal wound or mediastinal widening on radiography [30]. Cardiac decompensation is defined as a clinical syndrome in which a structural or functional disorder of the heart impairs the capacity of the ventricle to eject or fill with blood at physiologic filling pressures, therefore limiting the ability of the patient to exercise or carry out activities of daily living without symptoms of dyspnea or fatigue [31]. All outcomes are detailed in Textbox 2 [32,33].

**Primary and secondary outcome measures.**

**Primary study parameter**

- Detection of atrial fibrillation

**Secondary study parameters**

- Detection of sternal wound infection
- Detection of cardiac decompensation
- Time to detection of atrial fibrillation
- Time to detection of sternal wound infection
- Time to detection of cardiac decompensation
- Quality of life (five-level EuroQol five-dimension)
- Patient satisfaction of care
- Overall mortality
- Major adverse cardiac events: cardiac death, myocardial infarction, cardiac tamponade, ischemic stroke, or transient ischemic attack, defined as a transient episode of neurological dysfunction lasting for less than 1 hour, which is caused by focal brain or retinal ischemia without recent infarction on cerebral imaging [32].
- Readmission to either the cardiology or cardiothoracic surgery ward
- Total number of cardiology-related visits to the emergency department until 3 months after discharge
- Blood pressure control, which is defined as a systolic blood pressure of <140 mmHg and a diastolic blood pressure of <90 mmHg [33]
- Cost-effectiveness

### Questionnaires

All patients, both in the intervention and control groups, are asked to fill out a quality of life questionnaire (EQ-5D-5L) [34] and a short patient satisfaction questionnaire. The patients in the intervention group are also asked to fill out a short satisfaction questionnaire concerning the webcam consultation system and the used devices. The EQ-5D-5L questionnaire is used before surgery and 3 months after surgery. The satisfaction questionnaire is used 3 months after surgery.

### Sample Size and Statistical Analysis

An AF detection rate of 15% is expected in the historical group and 25% is expected in the intervention group [12,35]. Power calculation was performed using R software (R Foundation for Statistical Computing, Vienna, Austria) [36]. For the calculation, an α of .05 and a power of 0.90 were chosen. This yielded a sample size of 335 patients, which was increased by 9% to 365 patients in both the intervention and control groups to correct for expected loss to follow-up, leading to a total study size of 730 patients.

Patient information is analyzed according to the intention-to-treat principle, and a per protocol subanalysis is carried out. According to the unpublished data of *The Box* study, it is estimated that 33% of eligible patients will not be willing to take part in *The Box 2.0* study or will stop sending in measurements shortly after inclusion. Accordingly, to prevent selection bias, patients who do not wish to take home measurements with the devices of *The Box 2.0* are included in the intervention arm if they consent to the researchers using their data until 3 months after surgery.

With regard to the secondary endpoints, the diagnoses of sternal wound infection and cardiac decompensation are analyzed in the same way as the primary endpoint. The proportion of patients with controlled BP and the proportion of readmitted patients will be compared using a chi-square test. Logistic regression will be performed to correct for potential confounding variables. The scores of questionnaires (EQ-5D-5L and patient satisfaction questionnaires) and health care utilization will be compared using an independent *t* test. As this study is underpowered to detect differences between groups with respect to major adverse cardiac events (MACEs), only descriptive statistics of this outcome will be reported.

## Discussion

### Patient Selection

For this study, high-risk patients are excluded. The INTERMACS scale was developed to further categorize New York Heart Association Class IV patients [37]. We use this INTERMACS scale as a clinical tool to grade very ill patients, and we define high-risk patients as those with an INTERMACS score of 1 or 2. We exclude patients receiving a ventricular assist device, those with a possible life-threatening condition at the time of their surgery, such as active endocarditis and ventricular septal rupture, and those needing mechanical support with ECMO, IABP, or an Impella device at the time of the operation. This patient group is excluded from participation, as effects on outcomes, such as mortality, MACEs, and rhythm disturbances, would be incomparable between nonrandomized study groups owing to the high mortality and morbidity rates of high-risk patients [38-44]. Patients undergoing any other type of cardiac surgery are included.

### Internet Access

As of 2018, 98% of Dutch households have internet access; more than any other European country [45]. Therefore, generalizability of the results of this study might be limited in countries with low internet access percentages. The expected average age of subjects at the time of inclusion is 65 years. In the Netherlands, 84% of people aged 55 to 65 years, 68% of people aged 65 to 75 years, and 40% of people aged 75 years or older have internet access [46]. To prevent selection bias, an intention-to-treat design is chosen, in which patients without internet access are also enrolled in the intervention group.

### Comparability and Study Outcomes

To our knowledge, a study validating mHealth in cardiac surgery patients has not been performed. Pilot results from a randomized controlled trial performed by McElroy et al, which involved 27 intervention patients and 416 control patients who underwent cardiac surgery, showed no relevant results regarding hospital readmission rates when using a digital health kit, which included a tablet with a pulse oximeter, heart rate and blood pressure monitor, and weight scale [12]. In this previous study, the proportion of postoperative AF diagnosis did not significantly differ between the groups (29.6% [8/27] in the intervention group and 15.4% [64/416] in the control group; *P*=.90). This may be due to a correction for a difference at baseline, where having a history of AF differed between the groups (22.2% [6/27] in the intervention group and 11.3% [47/416] in the control group; *P*=.20) [12].

Currently available consumer mHealth devices may make ambulatory diagnosis of rhythm disturbances more accessible. As POAF does not always cause symptoms and thus could remain subclinical, we hypothesize that using these devices in patients after cardiac surgery will greatly increase the diagnosis of POAF.

Owing to the accessible nature of consumer mHealth devices, it is hypothesized that other complications that may arise after cardiac surgery, such as sternal wound infection and cardiac decompensation, will be diagnosed earlier as compared with conventional follow-up. Early detection of these abnormalities could lead to a diminished disease burden among patients and result in more outpatient treatment or shorter clinical treatment, causing increased cost-effectiveness. Moreover, as patients can read their own measurements and follow trends, they may feel more empowered with the use of the devices adopted in this study and may reach a higher quality of life.

### Definition of Atrial Rhythm Disturbances in mHealth

Defining POAF detected with mHealth devices is not possible according to current European or American guidelines, as mHealth is currently not a tool for diagnosis. Recent studies have found that, when using AF detection devices, prolonged episodes of AF, atrial flutter, or atrial tachycardia (lasting over 5 minutes in the MOST study and over 6 minutes in the ASSERT study) are independently associated with an increased risk of stroke [47,48]. However, short episodes of up to 20 seconds are not associated with an increased risk [49]. Devices of *The Box 2.0* do not measure heart rhythm in a continuous fashion, and thus, measurements should be compared with spot measurements involving a normal clinical ECG. We have therefore initiated a regional focus group with five cardiologists specialized in rhythm disturbances and two cardiac surgeons. This group defined POAF as AF shown on a CardioSecur ECG during the full 10 seconds when the heart rhythm is registered, which is diagnosed within 3 months after cardiac surgery. Other atrial rhythm disturbances, such as atrial flutter and atrial tachycardia, are addressed in the same fashion. For the treatment of diagnosed rhythm disturbances, the current European guidelines are followed [50].

### Conclusion

In summary, a study to investigate mHealth devices in cardiac surgery patients is presented. The first results are expected in September 2020.

## Appendix

Multimedia Appendix 1 Definitions of data irregularities.

## Abbreviations

AF: atrial fibrillation
BP: blood pressure
ECG: electrocardiogram
ECMO: extracorporeal membrane oxygenation
EQ-5D-5L: five-level EuroQol five-dimension
IABP: intra-aortic balloon pump
INTERMACS: Interagency Registry for Mechanically Assisted Circulatory Support
LUMC: Leiden University Medical Center
MACE: major adverse cardiac event
mHealth: mobile health
NP: nurse practitioner
POAF: postoperative atrial fibrillation
