# Supplementary material for: Use of Smart Technology for the Early Diagnosis of Complications After Cardiac Surgery: The Box 2.0 Study Protocol
Source: JMIR Res Protoc. 2020 Apr 21;9(4):e16326. doi: 10.2196/16326 (PMC7201318; doi:10.2196/16326)
Supplement: Multimedia Appendix 1 [file resprot_v9i4e16326_app1.docx]

**Multimedia Appendix 1.** Definitions of data irregularities.

| ***Blood pressure***   - Under 90 mmHg systolic or 60 mmHg diastolic: patient will remeasure and if the second measurement shows the same results, the patient will contact the physician. - Above 250 mmHg systolic or 130 mmHg diastolic: patient will remeasure and if the second measurement shows the same results, the patient will contact the physician. - Three consecutive days above 180 mmHg systolic or 110 mmHg diastolic: the patient will contact the physician. - Structurally above 140 mmHg systolic or 90 mmHg diastolic: at the next outpatient clinic visit (webcam or physical), this will be discussed with the patient. Treatment may be started.   ***Weight***   - Gaining 2 kilograms or more within 3 consecutive measurements, or with complaints of dyspnea or increasing peripheral edema: the patient will contact the physician. - Gaining 4 kilograms or more within 7 consecutive measurements: the patient will contact the physician. - Losing 3 kilograms or more within 7 consecutive measurements: the patient will contact the physician.   ***Temperature***   - Above 38,5 degrees centigrade: the patient will remeasure and if the second measurement shows the same results, the patient will contact the physician. - Below 35 degrees centigrade: the patient will remeasure and if the second measurement shows the same results, the patient will contact the physician. - Patients will also contact the physician when there are complaints of sternal redness, swelling, leakage, or general illness.   ***Pulse frequency***   - The Kardia shows a rhythm of < 45 beats per minute: the patient will make an ECG with the CardioSecur device and send it through to their physician. - The Kardia shows a rhythm of > 100 beats per minute: the patient will make an ECG with the CardioSecur device and send it through to their physician. - The Kardia shows an irregular rhythm, diagnosed as (possible) atrial fibrillation: the patient will make an ECG with the CardioSecur device and send it through to their physician. - The Kardia shows an undistinguishable rhythm: the patient will sit down and rest their arms on a chair or table. Then, another two Kardia registrations will be made. In case the rhythm stays undistinguishable, the patient will make an ECG with the CardioSecur device and send it through to their physician. - In case the patient experiences complaints of palpitations, dizziness or (near) collapse, the physician or alarm services will be contacted.   ***Saturation and respiration rate***   - In case a patient suffers from shortness of breath, the patient will contact the physician or the alarm services. - If the Masimo shows a blood oxygen saturation of 88% or lower: the patient will remeasure. With 2 or more consecutive measurements of a saturation of 88% or lower, the patient will contact the hospital. - The Masimo shows a blood oxygen saturation of 89 to 92%: the patient will remeasure. With 3 or more consecutive measurements of a saturation of 89 to 92%, the nurse practitioner will check the pulmonary history of the patient and contact the patient when deemed necessary. - The Masimo shows a respiration rate of 20 or higher: the patient will remeasure. With 2 or more consecutive measurements of a respiration rate of 20 or higher, the patient will contact the physician. |
| --- |

*Data irregularities which will be acted upon. All patients are carefully and repetitively instructed to contact their physician in case of complaints or if the measurements give a reason to do so.*
